# 淋巴瘤合并神经系统副肿瘤综合征11例临床分析

**DOI:** 10.3760/cma.j.issn.0253-2727.2022.04.008

**Published:** 2022-04

**Authors:** 冲 魏, 丹青 赵, 炎 张, 为 王, 薇 张, 道斌 周

**Affiliations:** 中国医学科学院、北京协和医学院北京协和医院血液内科，北京 100730 Department of Hematology, Peking Union Medical College Hospital, Chinese Academy of Medical Sciences & Peking Union Medical College, Beijing 100730, China

**Keywords:** 淋巴瘤, 副肿瘤综合征, 神经系统, 化疗, 免疫抑制治疗, Lymphoma, Paraneoplastic syndromes, Nervous system, Chemotherapy, Immunosuppression therapy

## Abstract

**目的:**

探讨淋巴瘤合并神经系统副肿瘤综合征（PNS）患者的临床特点、治疗及预后。

**方法:**

回顾性分析2012年1月至2021年5月于北京协和医院诊治的11例淋巴瘤合并PNS患者的临床资料。

**结果:**

11例患者中，男8例，女3例，中位发病年龄为62（16～74）岁。10例患者PNS症状先于淋巴瘤出现，出现PNS症状至诊断淋巴瘤的中位时间为4个月。11例患者中包括霍奇金淋巴瘤1例、B细胞型非霍奇金淋巴瘤8例、外周T细胞淋巴瘤2例。7例患者进行了肿瘤神经抗体的检测，2例阳性，分别为抗Ma2抗体和抗Yo抗体。11例患者中，PNS症状定位于中枢神经系统者3例，定位于周围神经系统者4例，定位于肌肉者3例。11例患者中8例在诊断淋巴瘤前采用以糖皮质激素为主的免疫抑制治疗，中枢神经系统病变及皮肌炎患者对免疫抑制治疗反应良好，周围神经病变患者激素治疗获益不明显。11例患者在诊断淋巴瘤后均采用针对淋巴瘤的化疗。其中9例行疗效评估，7例为完全缓解，1例为疾病稳定，1例为疾病进展。淋巴瘤获得完全缓解的患者PNS症状均获得极大改善。中位随访时间为42（4～95）个月。截至随访终止日期，11例患者中6例存活，3例失访，2例死亡，整组患者中位OS时间未达到。

**结论:**

PNS可累及神经系统的各个组成部分，可并发于各种类型的淋巴瘤。早期通过化疗达到肿瘤的完全缓解可使多数患者PNS症状改善。

## Abstract (en)

**Objective:**

To evaluate the clinical characteristics, treatment, and prognosis of patients with paraneoplastic neurological syndrome（PNS）associated with lymphoma.

**Methods:**

Between January 2012 and May 2021, the clinical data of 11 patients with lymphoma complicated with PNS treated at Peking Union Medical College Hospital were retrospectively reviewed.

**Results:**

Among the 11 patients（8 male and 3 female）, the median onset age was 61（range, 33–78）years. The symptoms of PNS preceded lymphoma in 10 patients. The median time from the onset of PNS to the diagnosis of lymphoma was 4 months. Of the 11 patients, one had Hodgkin's lymphoma, 8 had B-cell non-Hodgkin's lymphoma, and 2 had peripheral T-cell lymphoma. Seven patients were evaluated for onconeural antibody, of whom 2 were positive（1 for anti-Ma2 antibody and 1 for anti-Yo antibody）. Of the 11 patients, the PNS symptoms of 3 patients were located in the central nervous system, 4 were located in the peripheral nervous system, and 3 were located in the muscle. Eight of the 11 patients were treated with glucocorticoid-based immunosuppressive therapy before the diagnosis of lymphoma. Patients with central nervous system involvement and dermatomyositis responded well to glucocorticoid, whereas patients with peripheral neuropathy did not significantly benefit. All 11 patients were treated with chemotherapy after the diagnosis of lymphoma. The efficacy of chemotherapy was assessed in 9 patients, 7 cases achieved complete remission, 1 case was evaluated as stable disease, and 1 case was evaluated as disease progression. The PNS symptoms of the patients who achieved complete response were almost completely recovered. The median follow-up time was 42（range, 4–95）months. At the end of the follow-up period, 6 of the 11 patients survived, 3 were lost to follow-up, and 2 died. The median overall survival of the whole group was not reached.

**Conclusion:**

PNS can involve various parts of the nervous system and can be associated with different types of lymphoma. Through early diagnosis and treatment, the PNS symptoms could improve in most patients who achieve complete remission of lymphoma.

副肿瘤综合征是指发生在肿瘤患者中，在未出现肿瘤转移的情况下引起远隔的自身器官功能异常。副肿瘤综合征可影响体内的多种组织和器官，如所影响的远隔器官为神经系统，则称之为神经系统副肿瘤综合征（paraneoplastic neurological syndrome，PNS）。PNS非肿瘤直接转移或浸润所致，亦非感染、缺血、代谢、营养等因素或肿瘤相关治疗所致。淋巴瘤合并PNS较为罕见，国内仅有少量个案报道，尚无病例系列研究。由于PNS症状多先于淋巴瘤出现，且涉及多学科协作，多数患者的诊治过程艰难曲折。本研究回顾性分析了11例我院近10年确诊的淋巴瘤合并PNS患者的临床资料，以提高血液科医师对此疾病的认识。

## 病例与方法

1. 病例：回顾性分析2012年1月至2021年5月北京协和医院诊治的11例淋巴瘤合并PNS患者的临床资料。淋巴瘤的诊断参照2016年修订的世界卫生组织造血和淋巴系统肿瘤的分类标准[1]，由我院病理科医师经组织学和免疫组化检查确诊。PNS的诊断参照Graus等[2]在2004年提出的诊断标准，分为“确诊”和“拟诊”两个级别。

2. 临床资料：本研究收集的患者临床资料包括：性别、年龄、临床症状、体格检查、Ann Arbor分期、肿瘤神经抗体、脑脊液评估、MRI、肌电图及神经传导速度、肌肉活检、神经活检、治疗方式和疗效评估。肿瘤神经抗体的检测包括抗Hu、抗Yo、抗Ri、抗CV2、抗Ma2和抗amphiphysin抗体。

3. 疗效评估：淋巴瘤的疗效评估依据2007年Cheson淋巴瘤疗效评价修订标准，分为完全缓解（CR）、部分缓解（PR）、疾病稳定（SD）和疾病进展（PD）[3]。PNS的疗效评估主要依据神经系统查体及患者主观感受。

4. 随访：随访方式包括住院、门诊病例和电话随访。随访截止日期为2021年6月1日。总生存（OS）时间指自确诊淋巴瘤至因任何原因死亡或随访终止的时间。

5. 统计学处理：采用描述性统计分析，计量资料以中位数（范围）形式描述。

## 结果

1. 基本临床特征：11例淋巴瘤合并PNS患者的基本特征见表1。11例患者中，男8例，女3例，男女比例为2.7∶1。中位发病年龄为62（16～74）岁。其中10例患者PNS症状出现在淋巴瘤诊断之前，仅1例患者的PNS症状出现在淋巴瘤诊断之后。出现PNS症状至诊断淋巴瘤中位时间为4个月。11例患者中包括霍奇金淋巴瘤（Hodgkin's lymphoma，HL）1例、B细胞型非霍奇金淋巴瘤（B-NHL）8例，外周T细胞淋巴瘤2例。B-NHL中包括弥漫大B细胞淋巴瘤2例、套细胞淋巴瘤1例，其余为惰性B-NHL（滤泡性淋巴瘤1例、边缘区淋巴瘤1例、慢性淋巴细胞白血病1例、亚型未明2例）。11例患者中9例Ann Arbor分期为Ⅲ～Ⅳ期，IPI评分为中高危或高危者6例。参照Graus等[2]在2004年提出的PNS诊断标准，11例患者中8例PNS的诊断级别为“确诊”，3例为“拟诊”。

**表1:** 11例淋巴瘤合并神经系统副肿瘤综合征（PNS）患者的基本临床特征

例号	性别	年龄（岁）	PNS定位	PNS诊断	淋巴瘤类型	出现PNS症状至诊断淋巴瘤时间（月）	淋巴瘤疗效	PNS疗效	OS时间（月）	转归
1	男	62	中枢神经系统	边缘叶脑炎	滤泡性淋巴瘤	7	NA	失访，不详	6	失访
2	男	59	中枢神经系统	边缘叶脑炎	套细胞淋巴瘤	2	CR	完全恢复	24	存活
3	男	21	中枢神经系统	僵人综合征	霍奇金淋巴瘤	5	CR	完全恢复	30	存活
4	男	63	脊髓及周围神经	脊髓及周围神经脱髓鞘病变	惰性B细胞淋巴瘤	18	NA	部分改善	7	死亡
5	女	39	周围神经系统	感觉运动周围神经病	弥漫大B细胞淋巴瘤	4	CR	部分改善	42	失访
6	男	16	周围神经系统	感觉运动周围神经病	外周T细胞淋巴瘤，非特指	1	CR	部分改善	95	存活
7	男	73	周围神经系统	感觉运动周围神经病	慢性淋巴细胞白血病	-12^a^	SD	无改善	21	存活
8	男	74	周围神经系统	格林巴利综合征	外周T细胞淋巴瘤，非特指	4	PD	无改善	4	死亡
9	女	63	肌肉	多发性肌炎	惰性B细胞淋巴瘤	3	CR后复发	完全恢复	45	存活
10	男	63	肌肉	皮肌炎、肺间质病变	弥漫大B细胞淋巴瘤	15	CR	完全恢复	46	存活
11	女	42	肌肉	皮肌炎、肺间质病变	边缘区淋巴瘤	38	CR后复发	完全恢复，复发再现	69	失访

注：CR：完全缓解；SD：疾病稳定；PD：疾病进展；OS：总生存；NA：无数据；^a^ 诊断淋巴瘤时间为出现PNS症状前12个月

2. PNS的临床表现及实验室检查：依据神经系统症状的定位，PNS的临床特征分类阐述如下：①PNS症状定位于中枢神经系统者3例（例1～3），其中2例为边缘性脑炎，临床表现为精神异常和记忆力减退，1例伴全身癫痫发作；1例为僵人综合征，表现为四肢僵硬和痉挛。以上3例均完善脑脊液细胞学、免疫分型及头部MRI评估，未提示淋巴瘤累及中枢。②PNS症状定位于脊髓及周围神经者1例（例4），表现为下肢麻木无力和尿便障碍，结合影像学、脑脊液、肌电图评估，考虑为脊髓及周围神经脱髓鞘病变。③PNS症状定位于周围神经系统者4例（例5～8），其中3例为感觉运动周围神经病，表现为缓慢进展的以下肢为主的肌无力伴感觉异常；1例（例6）为格林巴利综合征，表现为急性进展的“上升性”肌无力，脑脊液出现蛋白-细胞分离现象。以上4例患者均行颈、腰椎MRI及PET/CT，未见神经根增粗、代谢升高等肿瘤累及表现。④PNS症状定位于肌肉者3例（例9～11），其中2例为皮肌炎（DM）伴肺间质病变，1例为多发性肌炎（PM）。11例患者中7例患者进行了肿瘤神经抗体检测，2例阳性，包括抗Ma2抗体（例1）和抗Yo抗体（例6）各1例。

3. 治疗及疗效：8例患者在明确诊断为淋巴瘤前以糖皮质激素治疗为主，部分联合免疫抑制剂和静脉注射丙种球蛋白（IVIG）治疗。淋巴瘤诊断后均采用针对淋巴瘤的化疗，部分联合靶向治疗、布鲁顿酪氨酸激酶抑制剂和自体造血干细胞移植。11例患者中9例行淋巴瘤的疗效评估，其中7例评估为CR，1例为SD，1例为PD。1例因感染死亡，1例化疗后失访未行评估。2例惰性B-NHL患者在获得CR后出现疾病复发。

不同类型的PNS对于免疫抑制治疗及淋巴瘤化疗的反应不同，具体如下：①3例（例1～3）PNS症状定位于中枢神经系统的患者经激素及IVIG治疗后PNS症状均有改善。2例（例2～3）在淋巴瘤化疗后PNS症状迅速缓解，另1例患者化疗后失访。②例4采用激素及利妥昔单抗治疗后PNS症状部分改善，但因骶尾部压疮感染暂停利妥昔单抗治疗，后神经系统症状急剧加重死亡。③PNS定位于周围神经系统的患者中2例（例5、例6）在淋巴瘤获得CR后神经系统症状明显改善，但未完全恢复。余2例（例7、例8）淋巴瘤尚未缓解，神经系统症状亦无改善。④3例肌炎/DM患者中2例（例10、例11）在诊断淋巴瘤前采用激素治疗，症状可完全缓解。另1例（例9）在淋巴瘤化疗后肌炎症状迅速缓解。1例在淋巴瘤复发前再次出现DM症状。

4. 转归：中位随访时间为42（4～95）个月。至随访截止，11例患者中6例存活，3例失访，2例死亡。2例患者的死亡原因分别为疾病进展和意识障碍合并肺部感染。11例淋巴瘤伴PNS患者的生存曲线见图1，整组患者中位OS时间未达到。

**图1:**
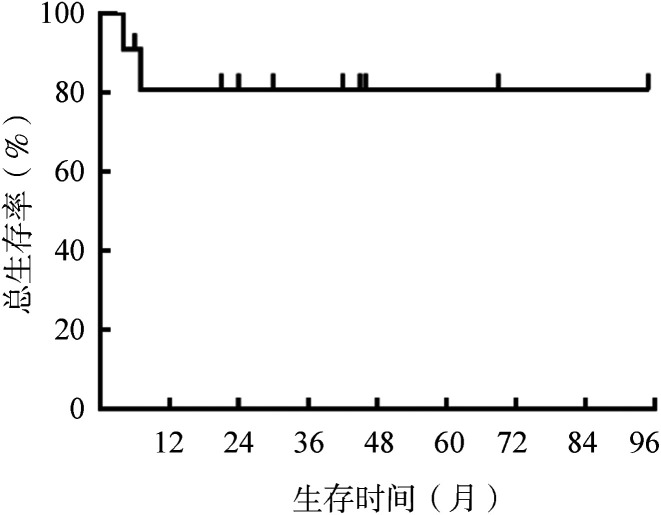
11例淋巴瘤伴神经系统副肿瘤综合征患者的总生存曲线

## 讨论

PNS指在未发生肿瘤直接浸润的情况下引起神经系统功能异常，PNS在实体肿瘤中的发病率为0.3％～1％，以小细胞肺癌最为常见[4]。PNS在淋巴瘤中相对罕见，多数为个案报道。目前最大规模的研究来自于PNS欧洲网络组织，该研究共纳入了2000-2008年注册于PNS欧洲网络数据库的53例淋巴瘤伴PNS患者，其中24例（45％）为HL，29例（55％）为B-NHL[5]。本研究11例淋巴瘤伴PNS的患者中仅1例为HL，余均为NHL。由于外周T细胞淋巴瘤好发于亚洲地区，本研究中2例患者为国外文献罕见报道的T细胞型NHL合并PNS。鉴于此疾病的罕见性，PNS在各类型淋巴瘤中的分布及发病率有待多中心研究或荟萃分析进一步探索。

Graus等[2]在2004年提出了PNS的诊断标准，分为“确诊”和“拟诊”两个级别。诊断流程图（图2）显示，“经典的PNS症状”和“特征性肿瘤神经抗体”是诊断PNS的重要依据。此外，抗肿瘤治疗后神经系统症状是否改善也具有重要的判断价值。该标准中“经典的PNS症状”包括脑脊髓炎、边缘叶脑炎、亚急性小脑变性、眼阵挛-肌阵挛、亚急性感觉神经元病、慢性胃肠假梗阻、Lambert-Eaton综合征和DM。“特征性肿瘤神经抗体”包括抗Hu、抗Yo、抗Ri、抗CV2、抗Ma2和抗amphiphysin抗体。本研究11例患者中4例临床表现为“经典的PNS症状”（边缘叶脑炎和DM各2例），2例肿瘤神经抗体检测为阳性（抗Ma2和抗Yo抗体各1例）。依据该诊断标准，8例PNS的诊断级别为“确诊”，3例为“拟诊”。

**图2:**
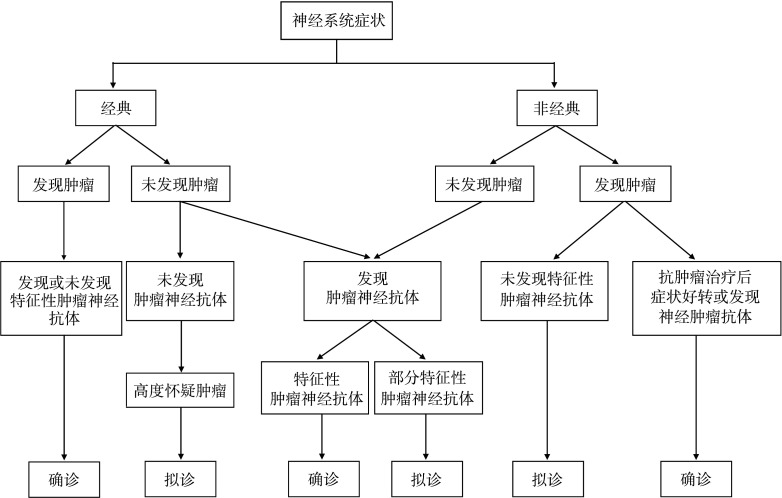
神经系统副肿瘤综合征的诊断流程图[2]

PNS的临床表现多样，可累及神经系统的各个组成部分，包括中枢神经系统、周围神经系统、神经肌肉接头和肌肉[6]–[7]。中枢神经系统受累的PNS以副肿瘤性小脑变性最常见，其次为边缘叶脑炎[5]–[8]。本研究中2例患者为NHL并发边缘叶脑炎，而国外研究报道较多的HL伴副肿瘤性小脑变性在本研究中未见。副肿瘤性周围神经病以周围神经脱髓鞘病变最为常见，主要包括慢性炎性脱髓鞘性多发性神经根神经病和格林巴利综合征，临床表现为对称性感觉及运动神经受累[6]–[7],[9]。本研究中副肿瘤性周围神经病变的特点概括为：肌力下降的程度较感觉异常更为突出，双侧基本对称，下肢重于上肢。DM/PM患者存在潜在恶性肿瘤的风险已被大量研究证实[10]–[11]。淋巴瘤伴DM/PM的研究发现约50％的DM/PM早于淋巴瘤出现，出现PM/DM症状至诊断淋巴瘤的中位时间为4.7个月[11]–[13]。本研究3例DM/PM患者中2例的肌炎症状早于淋巴瘤出现，最长达3年。1例患者在淋巴瘤复发前再次出现DM表现。因此，对于诊断为DM/PM的患者需充分筛查肿瘤，治疗中如出现DM症状反复，需警惕肿瘤的复发或进展。

PNS的治疗主要包括两个方面，一方面是针对肿瘤本身的治疗，无论何种类型肿瘤导致的PNS，最有效的治疗手段是尽早达到原发肿瘤的缓解；另一方面是针对PNS的免疫抑制治疗，主要包括糖皮质激素、免疫抑制剂、IVIG和血浆置换等[6]–[8]。多数患者在明确诊断肿瘤前采用免疫抑制治疗，在诊断肿瘤后开始化疗。本研究中7例淋巴瘤治疗有效患者的神经系统症状均获得极大改善，其中2例PNS定位于中枢神经系统的患者神经系统症状完全恢复，2例副肿瘤性周围神经病变的患者肌无力及感觉异常明显改善，但未完全恢复，3例DM/PM患者化疗后肌炎症状迅速缓解。总体而言，多数患者明确诊断淋巴瘤前可从免疫抑制治疗获益，尽早开始肿瘤化疗是改善PNS症状和减少神经系统后遗症的关键。远期预后方面，虽疾病早期部分患者因意识障碍、感染等并发症死亡，但淋巴瘤得到有效治疗后，淋巴瘤伴PNS患者可能获得与同类型淋巴瘤患者相近的远期生存。

综上所述，PNS的临床表现多样，可累及神经系统的各个组成部分，可并发于各种类型的淋巴瘤。当患者出现不明原因的神经系统异常，应警惕PNS可能，并进行肿瘤筛查。PNS的早期诊断可为神经系统功能恢复以及肿瘤治疗争取更多时间，提高肿瘤的治愈率。

## References

[1] Swerdlow SH, Campo E, Pileri SA (2016). The 2016 revision of the World Health Organization classification of lymphoid neoplasms[J]. Blood.

[2] Graus F, Delattre JY, Antoine JC (2004). Recommended diagnostic criteria for paraneoplastic neurological syndromes[J]. J Neurol Neurosurg Psychiatry.

[3] Cheson BD, Pfistner B, Juweid ME (2007). Revised response criteria for malignant lymphoma[J]. J Clin Oncol.

[4] Zekeridou A, Majed M, Heliopoulos I (2019). Paraneoplastic autoimmunity and small-cell lung cancer: Neurological and serological accompaniments[J]. Thorac Cancer.

[5] Briani C, Vitaliani R, Grisold W (2011). Spectrum of paraneoplastic disease associated with lymphoma[J]. Neurology.

[6] Graus F, Ariño H, Dalmau J (2014). Paraneoplastic neurological syndromes in Hodgkin and non-Hodgkin lymphomas[J]. Blood.

[7] Hagler KT, Lynch JW (2004). Paraneoplastic manifestations of lymphoma[J]. Clin Lymphoma.

[8] Blaes F (2021). Pathogenesis, diagnosis and treatment of paraneoplastic neurologic syndromes[J]. Expert Rev Neurother.

[9] Grisold W, Grisold A, Marosi C (2015). Neuropathies associated with lymphoma[J]. Neurooncol Pract.

[10] Airio A, Pukkala E, Isomäki H (1995). Elevated cancer incidence in patients with dermatomyositis: a population based study[J]. J Rheumatol.

[11] Zerdes I, Tolia M, Nikolaou M (2017). How can we effectively address the paraneoplastic dermatomyositis: Diagnosis, risk factors and treatment options[J]. J BUON.

[12] Marie I, Guillevin L, Menard JF (2012). Hematological malignancy associated with polymyositis and dermatomyositis[J]. Autoimmun Rev.

[13] 杨 华夏, 郭 静波, 肖 欣悦 (2017). 多发性肌炎/皮肌炎合并淋巴瘤的临床特点分析[J]. 基础医学与临床.

